# The ARID1A, p53 and ß-Catenin statuses are strong prognosticators in clear cell and endometrioid carcinoma of the ovary and the endometrium

**DOI:** 10.1371/journal.pone.0192881

**Published:** 2018-02-16

**Authors:** Marlene Heckl, Elisa Schmoeckel, Linda Hertlein, Miriam Rottmann, Udo Jeschke, Doris Mayr

**Affiliations:** 1 Institute of Pathology, Faculty of Medicine, Ludwig-Maximilians-University, Munich, Germany; 2 Department of Gynaecology and Obstetrics, Ludwig-Maximilians-University (LMU), Munich, Germany; 3 Munich Cancer Registry (MCR) of the Munich Tumour Centre (TZM), Institute for Medical Information Processing, Biometry, and Epidemiology (IBE), University Hospital of Munich, Ludwig-Maximilians-Universität (LMU), Munich, Germany; Universite du Quebec a Trois-Rivieres, CANADA

## Abstract

**Aim:**

The objective of this study was to evaluate the prognostic value of ARID1A, p53, p21, p16 and ß-Catenin in endometrioid and clear cell ovarian and endometrial carcinomas.

**Materials and methods:**

97 tumors were available for analysis of ARID1A, p53, p21, p16 and ß-Catenin with the techniques of tissue microarray and immunohistochemistry. 32 were ovarian carcinomas and 65 were endometrial carcinomas.

**Results:**

Endometrioid ovarian carcinomas showed negative staining for ARID1A (a) and p21 (b), aberrant expression of p53 (c) and p16 (d) and ß-Catenin positive nuclear expression (e) respectively in 19% (a), 100% (b), 28.6% (c), 52.4% (d) and 4.8% (e) of all cases. In the group of clear cell ovarian carcinomas it was 63.6% (a), 100% (b), 81.8% (c), 54.5% (d) and 0% (e). For endometrioid uterine carcinomas it was 75.7% (a), 94.9% (b), 30.5% (c), 52.1% (d) and 6.8% (e) and for clear cell uterine carcinomas it was 8.6% (a), 100% (b), 50% (c), 100% (d) and 0% (e). Survival analysis showed that negative expression of ARID1A, p53 aberrant expression and ß-Catenin nuclear positive staining are independent negative prognosticators in both, clear cell and endometrioid carcinoma, regardless of ovarian or uterine origin. Cox-Regression analysis showed them again as negative prognostic factors. Furthermore, we found a significant correlation between ARID1A and ß-Catenin expression in endometrioid uterine tumors.

**Conclusion:**

The analyzed gynaecological carcinoma showed a distinct expression scheme of proteins that are associated with tumor suppression. We may conclude that ARID1A, p53 and ß-Catenin are the strongest prognostic factors by analyzing a subgroup of tumor suppressor genes in clear cell and endometrioid subtypes of ovarian and endometrial cancer and may be used along with traditional morphological and clinical characteristics for prognosis.

## Introduction

The traditional histopathological classification of endometrial epithelial cancer, which was first proposed by Bokhman, includes type I tumors that are usually estrogen-dependent low-grade endometrioid cancers and type II tumors which are usually estrogen-independent high-grade serous or clear cell carcinomas [1]. While the first pathogenetic type has a frequency of 80–90% and is associated with highly or moderately differentiated tumors with a favorable prognosis, the second type has a frequency of only 10–20% and includes poorly differentiated tumors with a doubtful prognosis [1].

For ovarian epithelial cancer pathogenesis a less accepted paradigm exists suggesting to differ between type I and type II molecular profiles [2]. Type I tumors contain endometrioid, clear cell and low-grade serous carcinoma and mostly arise from atypical endometriosis or from borderline serous tumors [2,3]. Type II carcinomas include high-grade serous, which show typically a p53 mutation and, at least for patients with a BRCA mutation frequently arise from the fimbriated end of the fallopian tube via serous tubar intraepithelial carcinoma (STIC) [4,5].

Many studies have aimed to understand the cell origin and pathogenesis of these cancer subtypes in order to better diagnose and treat patients. Recently several studies suggested that the origin of clear cell and endometrioid carcinomas might derive from atypical endometriosis, which is believed to originate from the endometrium by retrograde menstruation [6–8]. The three-staged tumor grading system of endometrioid ovarian carcinoma is equivalent to the grading of endometrioid endometrial cancer and considers growth patterns and nuclear aplasia while there is no validated grading system for clear cell ovarian cancer, which are still classified as high-grade carcinoma [9].

Considerable interest has not only been generated in understanding the pathogenesis but also in the identification of factors that influence the prognosis of these tumors. Early diagnosis of epithelial ovarian and uterine cancer is critical for patient survival. Ovarian cancer has the highest mortality rate of the three main malignant tumors of the female reproductive system, with an overall 5-year survival rate of 45% [10]. Known prognostic factors of ovarian and endometrial carcinoma are histological subtype, tumor grading, International Federation of Gynecology and Obstetrics (FIGO) staging as well as estrogen receptor positivity for endometrial carcinoma and age, general condition and residual tumor for ovarian cancer [9,11]. Previous studies have explored molecular alterations in clear cell and endometrioid ovarian and endometrial tumors as additional prognostic factors, including changes in expression of ARID1A, p53, p21, p16 and ß-Catenin carcinoma.

ARID1A is a recently identified tumor suppressor participating in forming SWI/SNF chromatin complexes [12]. Somatic inactivating mutations of ARID1A and loss of ARID1A expression appear to be an early event in the development of most ovarian clear cell and endometrioid carcinomas as well as atypical endometriosis [13,14]. ARID1A is also frequently mutated and plays an important role in tumor progression in uterine endometrioid carcinoma [15,16].

p53 is a well-studied tumor suppressor gene that plays a key role in regulating the cell cycle. It is a principal mediator of growth arrest, senescence and apoptosis in response to a broad array of cellular damage [17]. The p53 wild-type protein directly induces the expression of the p21 protein which binds to a variety of cyclin-dependent kinases and inhibits their activity as well as regulates the repair of DNA and blocks its replication by inhibiting cell-cycle progression [18,19].

The p16 protein is also a tumor suppressor gene that, in response to various stresses, inhibits cyclin-dependent kinases and causes the arrest of the cell-cycle in G1 phase [20].

ß-Catenin however is the effector of the Wnt signaling pathway [21]. It accumulates in cell-cell junctions in cells not receiving the Wnt signal bound by a complex referred to as the destruction complex. When cells receive the Wnt signal it is stabilized, enters the nucleus and activates Wnt target genes [21]. Inappropriate activation of the Wnt pathway underlies many cancers including ovarian and uterine carcinoma, mostly endometrioid [22].

In this study the expression of ARID1A, p53, p21, p16 and ß-Catenin was determined in 97 tumors by performing immunohistochemistry to evaluate their prognostic value. Human tissue samples of the ovary and the uterus, obtained after surgical resection, were used to investigate the correlation between their expression and clinical parameters, including overall survival.

## Materials and methods

### Tumors and patients

This study assessed 97 patients who all underwent primary surgery between January 1, 1990 and December 31, 2001 in the Department of Gynaecology, Ludwig-Maximilians-University, Munich, Germany. A total of 59 cases were endometrioid uterine carcinoma, 6 clear cell uterine carcinoma, 21 endometrioid ovarian carcinoma and 11 clear cell ovarian carcinoma. The analyzed tissue samples were taken from the hospital archive of the Department of Pathology, Ludwig-Maximilians-University, Munich, Germany. In cooperation with the tumor register of Munich necessary data about the patients’ survival was available. The patients were staged and the tumors graded according to 1988 International Federation of Gynaecology and Obstetrics (FIGO) criteria [23]. Patients’ characteristics, *e*.*g*. age, FIGO stage, histological subtype and FIGO grade are shown in Table 1. Survival was taken from the date of confirmed histological diagnosis after primary surgery to the date of recurrence or last visit.

**Table 1:** Patients characteristics (N = 97).

Age (median)	61.0 (range 35–82)
Histopathology	
Clear cell uterine carcinoma	6 (6.2)
Endometrioid uterine carcinoma	59 (60.8)
Clear cell ovarian carcinoma	11 (11.3)
Endometrioid ovarian carcinoma	21 (21.6)
Tumor grading	
Grade 1	27 (27.8)
Grade 2	29 (29.9)
Grade 3	41 (42.3)
FIGO-staging	
I	24 (24.7)
II	20 (20.6)
III	24 (24.7)
IV	29 (29.9)

### Ethical approval

All patients´ data were fully anonymized, and the study was performed according to the standards set in the Declaration of Helsinki 1975. All tumor tissue used was leftover material that had initially been collected for histopathological diagnostics. All diagnostic procedures had already been fully completed when samples were retrieved for the study. The current study was approved in writing by the Ethics Committee of the Ludwig Maximilians University, Munich, Germany (approval number 449–14). Authors were blinded for clinical information during experimental analysis.

### Sampling and tissue microarray construction of ovarian and uterine cancer tissue

New samples from the original slides of tumors were taken and representative areas of tumor tissues were selected. Three core biopsies from each specimen were removed and attached on tissue microarrays. The presence of tumor tissue on the arrayed samples was verified by a pathologist.

### Immunohistochemistry and interpretation

Serial sections of the recipient tissue microarray paraffin blocks were cut at 2–3 μm, deparaffinized with xylene, and rehydrated through a series of graded alcohols. The immunostaining procedure was performed using an automated stainer (Benchmark® XT, Ventana). The following monoclonal primary antibodies were used: ARID1A/BAF250a Rabbit mAb (New England Biolabs GmbH) directed against ARID1A protein, p53 Ab-5 (Thermo Scientific) directed against 53, p16-Arc (p16INK4a, CINtec® Histology) directed against p16, p21 Cip (CDKN1A) directed against p21 and ß-Catenin Mouse IgG-1 (Roche, Ventana, ready to use) directed against ß-Catenin. All staining’s were performed at the Department of Pathology, Ludwig-Maximilians-University, Munich.

**Fig 1:**
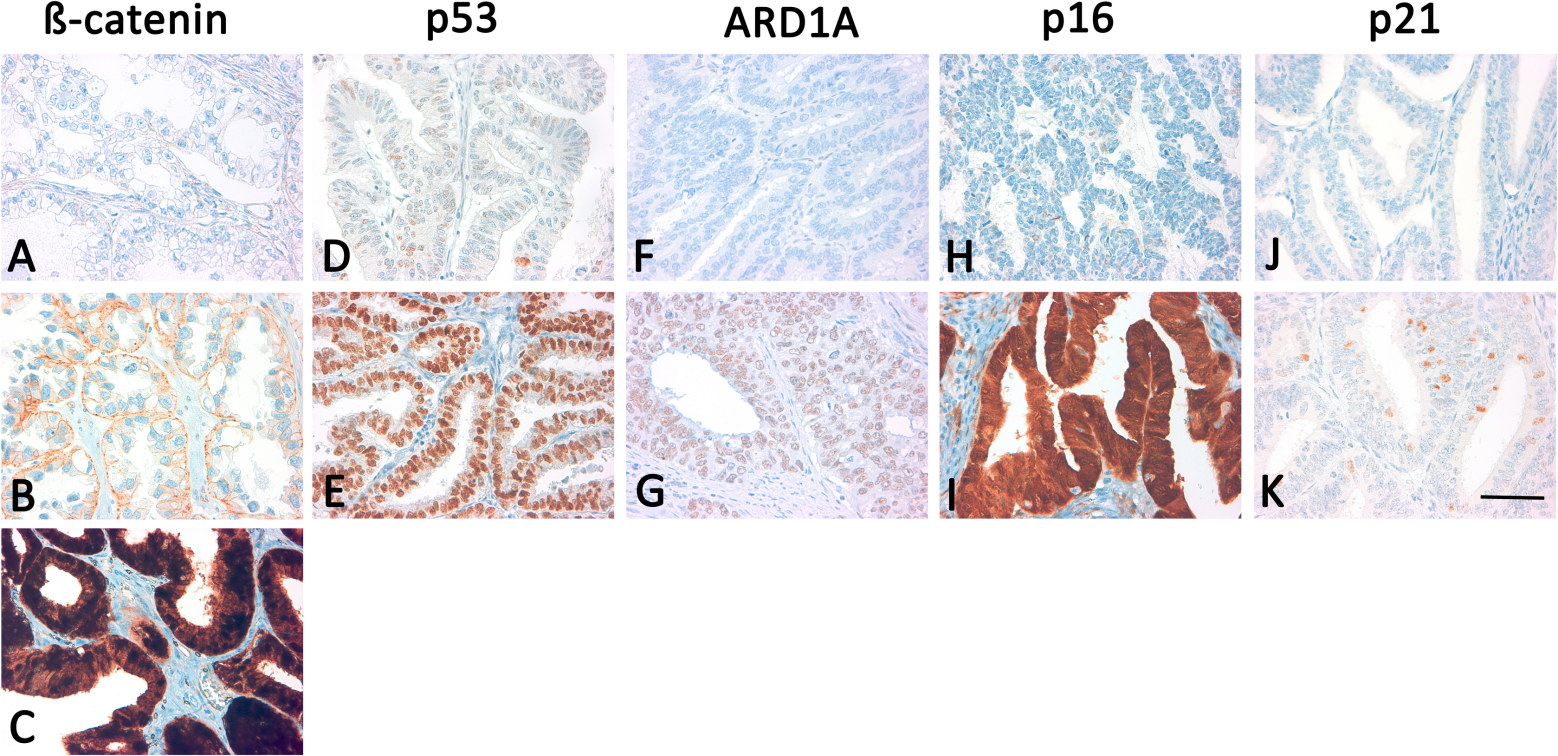
**Immunohistochemical stainings for ß-Catenin, p53, ARID1A, p16 and p21 in clear-cell (A and B) and endometrioid (C—K) carcinomas**. Focal, very weak membranous (n-m-) (A), membranous (n-m+) (B) and nuclear (n+m+) (C) expression of β-catenin. Regulated (D) and aberrant (E) expression of p53. Preserved expression of ARID1A (F) and loss (G) of ARID1A expression. Negative staining (H) and positive (I) staining for p16. Negative (J) and positive staining (K) of p21. 400 × magnification was used for all pictures; scale bar (K) refers to 60 μm for all images.

The IHC stains were evaluated by the authors (MH and DM) in a double-blind process using the immunoreactive Remmele score (IRS) [24]. The quantity of cells stained was scored as: 0 = no staining, 1 = 1–10%, 2 = 11–50%, 3 = 51–80% and 4 = >81% staining. The IRS was rendered as a product of the scores obtained for staining intensity (0 = no expression, 1 = weak expression, 2 = moderate expression, 3 = strong expression) and quantity. A total score of 0–2 was considered negative, 3–5: weak, 6–8: moderate and 9–12: strong immunoreactivity. ARID1A and p21 were dichotomized into negative (0–2 points in IRS) and positive cases (3–12 points in IRS) and p53 and p16 were dichotomized into no expression/overexpression (= aberrant) and normal expression (= regulated). Weak to moderate immunoreactivity (3–8 points in IRS) was considered p53/p16 normal expression. Strong and diffuse nuclear p53/p16 immunoexpression (9–12 points in IRS) or complete absence of p53/p16 staining (0–2 points in IRS) was interpreted as likely indicating a p53/p16 gene mutation. The presence of rare weakly positive nuclear staining is a pattern that is commonly associated with wild type p53 and can be found in normal ovarian and uterine tissues [25]. ß-Catenin was classified in 3 groups of ß-Catenin nuclear negative (0–2 points in IRS) and membrane positive (n-m+) staining (3–12 points in IRS), nuclear negative (0–2 points in IRS) and membrane weak (3–6 points in IRS) or negative staining (n-m-) (0–2 points in IRS) and ß-Catenin nuclear and membrane/cytoplasm positive (n+m+) staining (3–12 points in IRS) (Fig 1).

### Statistical analysis

For statistical analysis the SPSS Statistics Version 23 (SPSS Inc., Chicago, IL, USA) was used. For testing proportional differences in univariate analysis the Pearson’s Chi-square test or Fisher’s exact test for qualitative variables and unpaired t-test for quantitative normally distributed variables was applied. For normally distributed variables in several groups the single-factor variance analysis was used. The survival curves were generated by using the Kaplan Meier technique and differences between these curves were tested by the log-rank test. All tests were two-sided and the level of statistical significance was accepted at *p ≤* 0.05. For multivariate analyses the Cox regression model was used with tumor-related death as the endpoint.

## Results

### Results from immunohistochemistry

#### ARID1A

**Fig 2:**
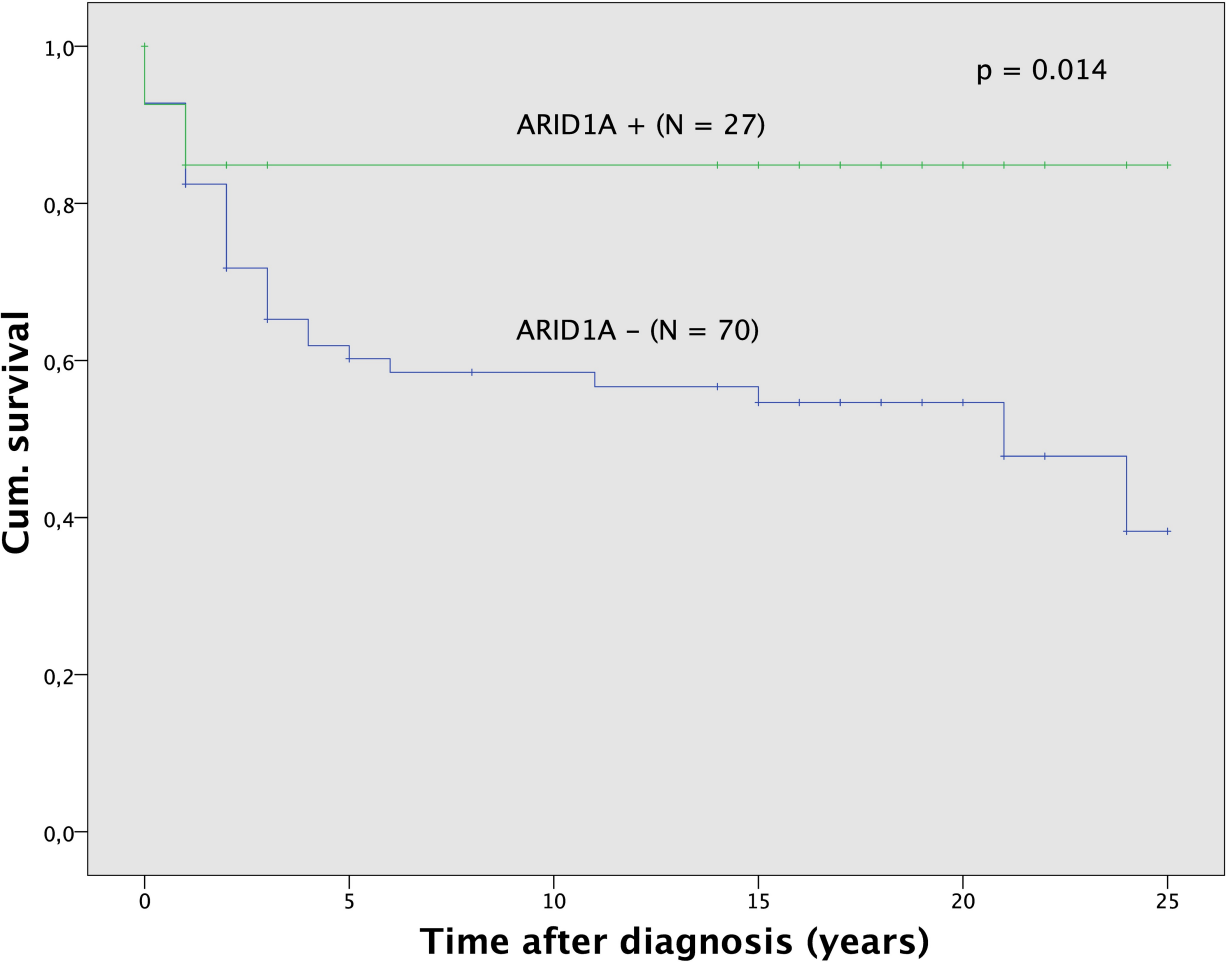
Survival analysis for ARID1A. Survival was better for the subgroup of patients with ARID1A positive tumors (N = 27) compared to the subgroup with ARID1A negative tumors (N = 70).

ARID1A nuclear positive staining was observed in 27 (27.8) out of 97 tumors. The ARID1A status was significantly (p < 0.001) related to histological subtype (Table 2). Positive nuclear staining seen more frequently in endometrioid tumors and it was infrequent in clear cell carcinoma. The ARID1A status alone was not associated with tumor grade (p = 0.581) or age (p = 0.369), but it was related statistically significantly to FIGO stage (p < 0.001). Positive nuclear staining for ARID1A was seen more frequently in FIGO I tumors while FIGO III and IV tumors were mostly ARID1A negative (S1 Fig). Survival analysis (Fig 2) demonstrated significant (p = 0.014) differences for patients according to the ARID1A status. Patients with ARID1A negative tumors had a 5-year survival of 60.2% compared with 84.9% survival for those with ARID1A positive tumors. Significantly better survival in the subgroup of ARID1A positive tumors could also be observed for sole analysis of all endometrioid (p = 0.039) and all ovarian carcinoma (p = 0.008) as well as tumors of grading G3 (p = 0.028).

**Table 2:** Status of ARID1A, p53, p21, p16 and ß-Catenin according to histological subtypes (N = 97).

Expression N (%)	ARID1A –	ARID1A +	P53 -/+++	P53 +	P16 -/+++	P16 +	P21 –	P21 +	ß-Catenin n-m+	ß-Catenin n-m-	ß-Catenin n+m+
	70 (72.2)	27 (27.8)	36 (37.1)	61 (62.9)	48 (49.5)	49 (50.5)	94 (96.9)	3 (3.1)	64 (66.0)	28 (28.9)	5 (5.2)
Histology											
Clear cell uterine	6 (8.6)	0 (0)	3 (8.3)	3 (4.9)	6 (12.5)	0 (0)	6 (6.4)	0 (0)	1 (1.6)	5 (17.9)	0 (0)
Endometrioid uterine	53 (75.7)	6 (22.2)	18 (50)	41 (67.2)	25 (52.1)	34 (69.4)	56 (58.3)	3 (3.1)	42 (65.6)	13 (46.4)	4 (80)
Clear cell ovarian	7 (10.0)	4 (14.8)	9 (25)	2 (3.3)	6 (12.5)	5 (10.2)	11 (11.5)	0 (0)	6 (9.4)	5 (17.9)	0 (0)
Endometrioid ovarian	4 (5.7)	17 (63.0)	6 (16.7)	15 (24.6)	11 (22.9)	10 (20.4)	21 (21.9)	0 (0)	15 (23.4)	5 (17.9)	1 (20)
P-value	< 0.001	< 0.001	0.008	0.008	0.049	0.049	0.756	0.756	0.07	0.07	0.07

#### p53

**Fig 3:**
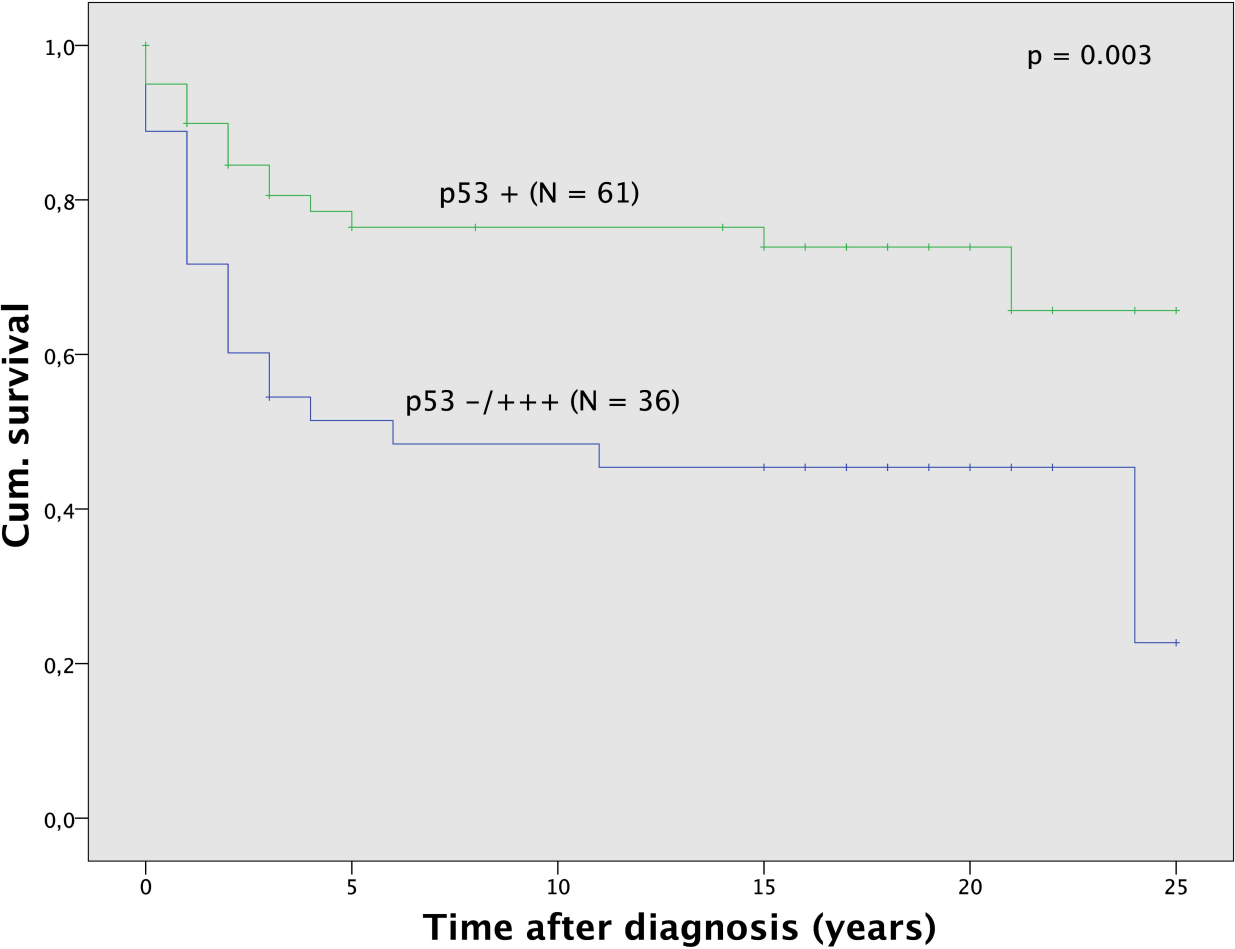
Survival analysis for p53. Survival was better for the subgroup of patients with p53 positive tumors (N = 61) compared to the subgroup with p53 negative/overexpression tumors (N = 36).

p53 overexpression or complete absence was seen in 36 (37.1) out of 97 carcinoma. The status of p53 was significantly (p = 0.008) associated with histological subtype (Table 2). p53 overexpression or complete absence was seen mostly in clear cell tumors, but infrequent in endometrioid carcinoma. The p53 status was not related to age (p = 0.487) or FIGO stage (p = 0.081), but it was associated significantly (p < 0.001) with tumor grade. Overexpression or no expression of p53 was most frequently seen in poorly differentiated (G3) clear cell and endometrioid tumors and infrequent in G1 and G2 endometrioid tumors (S1 Table). Survival analysis (Fig 3) showed significant (p = 0.003) differences for patients according to p53 status. Patients with overexpression or complete absence of p53 in their tumors had a 5-year survival rate of 51.5% compared to 76.5% survival for those with positive p53 expression. Significantly better survival in the subgroup of p53 regulated tumors could also be observed for sole analysis of all clear cell (p = 0.020) and all uterine carcinoma (p = 0.012) as well as tumors of grading G1 and G2 (p = 0.036) and G3 (p = 0.011) and tumors with FIGO staging 0, I and II (p = 0.012).

#### p16

p16 overexpression or complete absence was seen in 48 (49.5) out of 97 tumors. The p16 status was significantly (p = 0.049) related to histological subtype (Table 2). p16 overexpression or no expression was seen more frequently in clear cell tumors and it was infrequent in endometrioid carcinoma. The p16 status alone was not associated with tumor grade (p = 0.749), age (p = 0.359), FIGO stage (p = 0.645) or survival (p = 0.436).

#### p21

p21 nuclear positive staining was confined to the nucleus and observed in 3 (5.1%) out of 97 carcinoma, which were all endometrioid uterine tumors. The p21 status was not related to histological subtype (p = 0.756), grading (p = 0.114), age (p = 0.673), FIGO stage (p = 0.433) or survival (p = 0.153).

#### ß-Catenin

**Fig 4:**
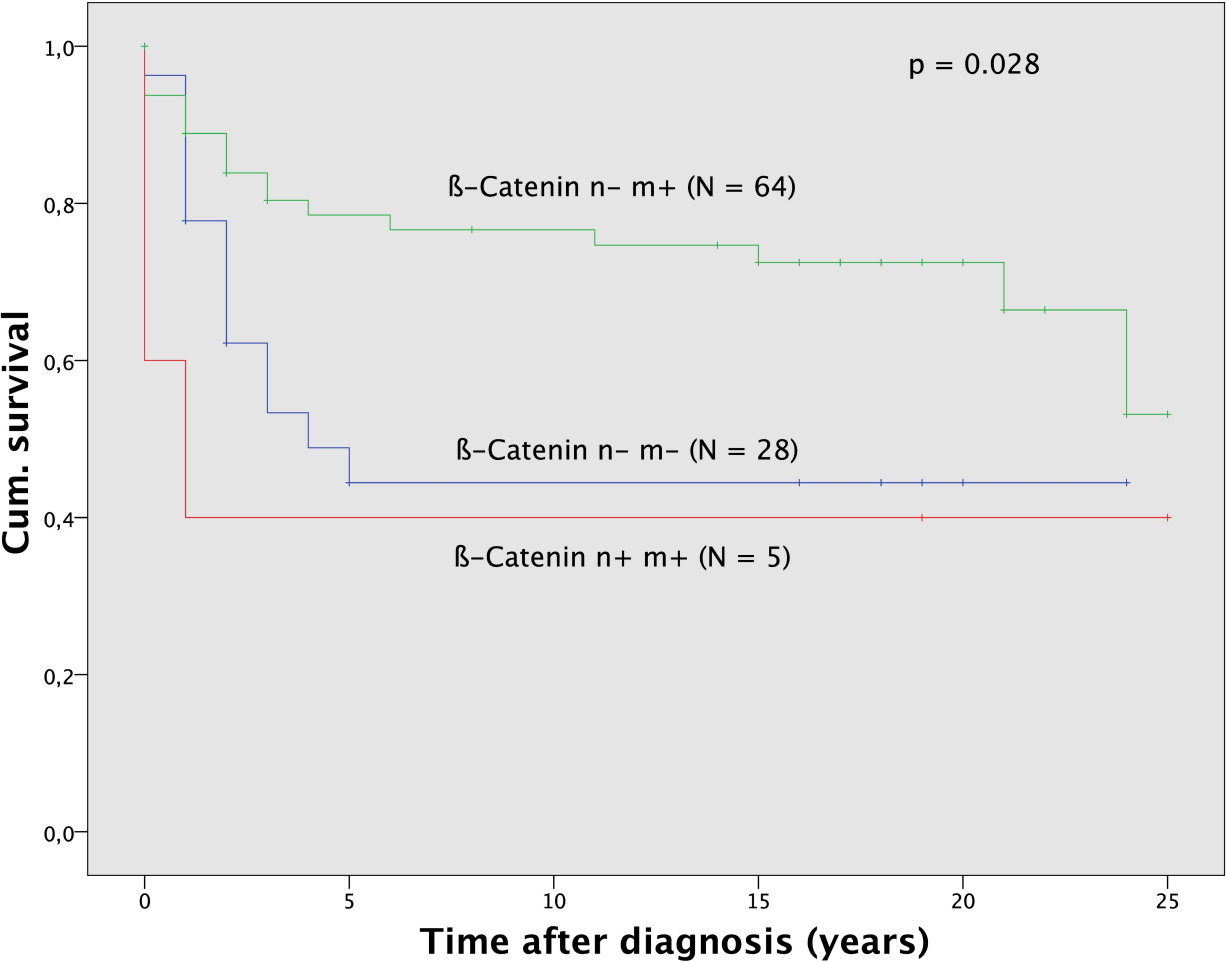
Survival analysis for ß-Catenin. Survival analysis for ß-Catenin. Survival was better for the subgroup of patients with ß-Catenin n-m+ tumors (N = 64) compared to the subgroup ß-Catenin n-m- (N = 28) and ß-Catenin n+m+ tumors (N = 5).

ß-Catenin nuclear negative and positive membrane staining (n-m+) was observed in 64 (66.0), ß-Catenin nuclear and membrane negative (n-m-) staining in 28 (28.8) and ß-Catenin positive nuclear and membrane/cytoplasm staining (n+m+) in 5 (5.2) out of 97 tumors. The ß-Catenin status was significantly (p = 0.07) related to histological subtype (Table 2). ß-Catenin n-m+ staining was more frequently seen in endometrioid tumors and it was infrequent in clear cell carcinoma. ß-Catenin n+m+ staining was only observed in endometrioid tumors, mostly in endometrial cancer. The ß-Catenin status was not related to age (p = 0.483) or FIGO stage (p = 0.750). It was significantly (p = 0.046) associated with tumor grade. Thus ß-Catenin n+m+ staining and n-m- staining was mostly seen in G2 and G3 tumors whereas positive membrane staining alone was almost evenly spread among all grading. Survival analysis (Fig 4) demonstrated significant (p = 0.028) differences for patients according to ß-Catenin status.

Patients with n-m- staining for ß-Catenin in their tumors had a 5-year survival rate of 44.4% and patients with n+m+ staining had a 5-year survival rate of 40,0% compared to 78.5% survival for those with n-m+ expression. Significantly better survival in the subgroup of ß-Catenin n-m+ stained tumors could also be observed for sole analysis of all endometrioid tumors (p = 0.031) and uterine carcinoma (p = 0.014) as well as tumors of grading G3 (p = 0.036) and tumors with FIGO staging III and IV (p = 0.021).

### Relationship between ARID1A/ß-Catenin status, and ß-Catenin/p16 status and their association to clinico-pathological data

Correlation between the expressions of the five different proteins was tested by using Pearsons chi-square test (Table 3). A low significant correlation was found between ARID1A and ß-Catenin with a corrected coefficient of contingency of C_cor_ = 0.282 (p = 0.045) and ß-Catenin and p16 with a corrected coefficient of contingency of C_cor_ = 0.282 (p = 0.045). Further the differences between the expressions of ARID1A/ß-Catenin and ß-Catenin/p16 were investigated based on different clinical parameters, including age, histological subtype, grading and FIGO staging. While the ARID1A/ß-Catenin status was not significantly related to age (p = 0.697), a significant correlation to histological subtype (p < 0.001), grading (p = 0.018) and FIGO staging (p = 0.014) was observed (Table 4). ARID1A negative staining and simultaneous ß-Catenin n+m+ staining was only seen in endometrioid uterine tumors. ARID1A negative staining and simultaneous ß-Catenin n-m- staining was also observed mostly in endometrioid endometrial cancer. This staining combination was found to be mostly in tumors with grading G3 and FIGO staging IV. ARID1A positive staining and simultaneous ß-Catenin n-m+ staining were mostly seen in endometrioid ovarian carcinoma and the tumors belonged mostly to FIGO staging I.

**Table 3:** Correlation among ARID1A/ß-Catenin and ß-Catenin/p16 in endometrioid and clear cell ovarian and uterine tissues.

	ß-Catenin n-m-/n+m+	ß-Catenin n-m+	
N (%)	33 (34.0)	64 (66.0)	N (%)
ARID1A -	28	42	70 (72.2)
ARID1A +	5	22	27 (27.8)
C_corr_ = 0.282, p-value: 0.045			97
P16 –o +++	21	27	48 (49.5)
P16 +	12	37	49 (50.5)
C_corr_ = 0.282, p-value: 0.045			97

C_corr_, corrected coefficient of contingency

**Table 4:** Status of ARID1A/ß-Catenin according to histological subtype, grading and FIGO staging.

Expression N (%)	ARID1A +/ ß-Catenin n-m+	ARID1A +/ ß-Catenin n+m+	ARID1A -/ ß-Catenin n-m-	ARID1A -/ ß-Catenin n-m+	ARID1A -/ ß-Catenin n+m+
	25 (25.8)	2 (2.1)	25 (25.8)	42 (43.3)	3 (3.1)
Histological subtype					
Clear cell uterine	0 (0)	0 (0)	5 (20)	1 (2.4)	0 (0)
Endometrioid uterine	5 (20)	1 (50)	13 (52)	37 (88.1)	3 (100)
Clear cell ovarian	4 (16)	0 (0)	5 (20)	2 (4.8)	0 (0)
Endometrioid ovarian	16 (64)	1 (50)	2 (8)	2 (4.8)	0 (0)
P-value < 0.001					
Grading					
G1	7 (28)	1 (50)	3 (12)	16 (38.1)	0 (0)
G2	5 (20)	1 (50)	6 (24)	16 (38.1)	1 (33.3)
G3	13 (52)	0 (0)	16 (64)	10 (23.8)	2 (67.7)
P-value = 0.018					
FIGO staging					
FIGO I	13 (52)	1 (50)	4 (16)	6 (14.3)	0 (0)
FIGO II	6 (24)	1 (50)	4 (16)	9 (21.4)	0 (0)
FIGO III	4 (16)	0 (0)	7 (28)	12 (28.6)	1 (33.3)
FIGO IV	2 (8)	0 (0)	10 (40)	15 (35.7)	2 (67.7)
P-value = 0.014					

The ß-Catenin/p16-status showed no significant correlation to the parameters mentioned above (age (p = 0.750), histological subtype (p = 0.189), grading (p = 0.379) and FIGO staging (p = 0.613)).

### Multivariate analysis

In a multivariate Cox regression analysis with tumor-related death as the end point significant and independent prognostic factors were ARID1A status with hazard ratio (HR) of 3.359, p53 status with HR of 3.408, ß-Catenin status with HR of 2.251 and ARID1A/ß-Catenin status with HR of 2.209 (Tables 5–8). An HR of 2.209 for concomitant ARID1A negativity and ß-Catenin n-m-/n+m+ staining of tumors versus other combinations of ARID1A/ß-Catenin meant a 2.209 fold increased risk for tumor-related death for patients who belonged to the first subgroup. Similarly, an HR of 3.359 for ARID1A negative tumors versus ARID1A positive tumors, an HR of 3.408 for p53 aberrant expression versus p53 regulated expression and an HR of 2.251 for ß-Catenin weak/negative membranous and positive nuclear expression versus ß-Catenin positive membranous expression meant a 3.359, 3.408 respectively 2.251 fold increased risk for tumor-related death for patients who belonged to the first subgroups.

**Table 5:** Multivariate Cox regression analysis with tumor related death as endpoint for ARID1A.

Variable	Hazard Ratio	95% Confidence Interval	P-Value	n
Age (years)	0.990	0.959–1.021	0.514	97
Histological subtype (endometrioid *vs*. clear cell carcinoma)	2.110	0.942–4.728	0.070	97
Grading (G1+G2 *vs*. G3 tumors)	1.064	0.524–2.162	0.864	97
FIGO stage (I+II *vs*. III + IV tumors)	1.079	0.533–2.186	0.833	97
ARID1A (+ vs. -)	3.359	1.152–9.792	0.026	97

**Table 6:** Multivariate Cox regression analysis with tumor related death as endpoint for p53.

Variable	Hazard Ratio	95% Confidence Interval	P-Value	n
Age (years)	0.987	0.956–1.019	0.426	97
Histological subtype (endometrioid *vs*. clear cell carcinoma)	1.605	0.716–3.599	0.251	97
Grading (G1+G2 *vs*. G3 tumors)	1.957	0.903–4.239	0.194	97
FIGO stage (I+II *vs*. III + IV tumors)	1.589	0.790–3.196	0.194	97
p53 (+ vs.—o+++)	3.408	1.567–7.415	0.002	97

**Table 7:** Multivariate Cox regression analysis with tumor related death as endpoint for ß-Catenin.

Variable	Hazard Ratio	95% Confidence Interval	P-Value	n
Age (years)	0.993	0.961–1.026	0.674	97
Histological subtype (endometrioid *vs*. clear cell carcinoma)	1.743	0.774–3.928	0.180	97
Grading (G1+G2 *vs*. G3 tumors)	1.430	0.691–2.957	0.335	97
FIGO stage (I+II *vs*. III + IV tumors)	1.249	0.633–2.511	0.532	97
ß-Catenin (n-m+ vs. n-m-/n+m+)	2.251	1.096–4.625	0.027	97

**Table 8:** Multivariate Cox regression analysis with tumor related death as endpoint for ARID1A/ß-Catenin status.

Variable	Hazard Ratio	95% Confidence Interval	P-Value	n
Age (years)	0.992	0.960–1.024	0.614	97
Histological subtype (endometrioid *vs*. clear cell carcinoma)	1.678	0.736–3.822	0.218	97
Grading (G1+G2 *vs*. G3 tumors)	1.459	0.696–3.058	0.317	97
FIGO stage (I+II *vs*. III + IV tumors)	1.198	0.593–2.421	0.614	97
Others vs. ARID1A-/ß-Catenin n-m-/ß-Catenin n+m+	2.209	1.031–4.734	0.041	97

## Discussion

Endometrial cancer can be subdivided into two histological subtypes, the estrogen-associated type I which includes endometrioid carcinomas and the estrogen-independent type II which comprises mostly high-grade serous and clear cell carcinoma [1]. The type II carcinoma is known to metastasize more often and to have a worse survival. A less accepted paradigm for ovarian cancer also differs between type I tumors which include endometrioid, clear cell and low-grade serous carcinoma and type II tumors containing p53-mutated high-grade serous carcinoma [2].

Many biomarkers, including different tumor suppressors such as ARID1A, p53, p21, p16 and ß-Catenin, were discovered to be changed in expression in endometrioid and clear cell subtypes in recent years [26–29]. The aim of this study was to evaluate their prognostic value, particularly in the synopsis of all the above mentioned factors, by using immunhistochemical methods.

Ovarian clear cell carcinoma usually showed p53 overexpression or no expression (81.8%), while ovarian endometrioid carcinoma mostly was observed to have positive p53 expression (71.4%). Both subtypes consisted in equal parts of p16 overexpression/no expression or positive expression and all stained negatively for p21. These findings confirm the results of various studies [30–32], which indicate that most of the ovarian clear cell tumors show p53 mutations, while most of endometrioid tumors do not. There are several studies showing that p16 is overexpressed in high-grade serous ovarian carcinoma compared with other morphologic types of ovarian cancer [33], whereas inactivation of the gene was observed in 3 out of 9 endometrioid and 1 out of 4 clear cell ovarian carcinomas [34]. Another study explored the expression of p21 in endometrioid and clear cell ovarian carcinoma and found p21 negativity in 59.5% (endometrioid) and 31.2% (clear cell) [35].

For endometrial carcinoma we can report that clear cell subtypes consisted in equal parts of p53 overexpression/no expression or positive expression and all showed p16 overexpression/no expression while endometrioid subtypes mostly were found to have positive p53 (69.5%) and p16 (69.4%) staining. Both subtypes were stained mostly p21 negative. In several studies, the p53 mutation prevalence among clear cell uterine carcinoma ranges from 28.5% to 76.9% [36,37].

For endometrioid uterine carcinoma however, most of the tumors appear to have no p53 mutation [38], which is in agreement with our results. In endometrioid uterine carcinoma the expression pattern of p16 is typically described as weakly positive unlike the strong expression generally seen in endocervial adenocarcinomas of the usual type [39]. However, another study found up to 33% overexpression and 65% no expression in clear cell endometrial carcinoma and different p16 expression in endometrioid endometrial carcinoma according to FIGO grade [40]. In FIGO grade 1 and 2 endometrioid subtypes were found to be mostly stained p16 negative while in FIGO grade 3 about 25% turned out to be p16 positive. For p21 expression in endometrial cancer some studies showed about 54% p21 negativity, increasing with older age and current smoking [41]. In contrast to these results other studies figured out that p21 seems to be mostly positively expressed in endometrial cancer [26]. The contradictory findings related to p16 and p21 may be due to different staining protocols, cut-off values for p16 and p21 expression, and characteristics of the study populations examined.

In this analysis the ß-Catenin expression of ovarian and uterine tumors was determined. ß-Catenin mutations are particularly common in endometrioid ovarian and uterine cancer [22]. However, their prevalence ranges widely from 16–54% across the several studies [42]. It could be confirmed that ß-Catenin n+m+ staining was only seen in endometrioid tumors, mostly uterine.

ARID1A has been recently classified as a novel tumor suppressor, which regulates p53-controlled genes [43]. Reduced ARID1A expression is mostly induced by nonsense mutations as well as insertions and deletions in the gene-coding region, which lead to mRNA decay or sequence truncation [44]. It has been published that promoter hypermethylation may also be responsible for the loss of ARID1A expression [45]. ARID1A mutations are frequently in various tumors including gastric cancer, colorectal cancer, breast cancer, lung cancer and gynaecological cancer [46]. High rates of ARID1A mutations were observed in 46–57% of ovarian clear cell carcinomas, 30% of ovarian endometrioid carcinomas and 40% of uterine endometrioid carcinomas [15,47,48]. In this study, we also demonstrated that negative expression of ARID1A was common in clear cell (63.6%) and endometrioid (19%) ovarian tumors as well as clear cell (100%) and endometrioid (89.8%) uterine cancer. Moreover, we determined a significant relationship between ARID1A loss and higher FIGO stages which is similar to previous studies [49,50].

No study could be found that examined the combined ARD1A/ß-Catenin status of ovarian and uterine tumors yet. However, few studies indicate that there is an association between the two tumor suppressor genes. ARID1A silencing seems to be inducing a subcellular redistribution of β-catenin from the plasma membrane to the cytoplasm and nucleus in gastric cancer, which was found to be significantly associated with worse clinical prognosis [51]. Another study demonstrated that the chromatin-remodeling factor ARID1B, forming the BAF complex together with ARID1A, represses the Wnt/ß-Catenin signaling pathway indicating this might contribute to cancer through deregulation of developmental and oncogenic pathways [52]. It also suggests ARID1A might operate in a similar fashion as ARID1B in Wnt/β-Catenin pathway. In this study a significant correlation between ARID1A and ß-Catenin could be shown. Concomitantly ARID1A negative staining and ß-Catenin n+m+ staining respectively ß-Catenin n-m- staining was usually seen in endometrioid uterine cancer with poor grading (G2 and G3) and poor FIGO staging (FIGO III and FIGO IV). In a multivariate Cox Regression analysis this staining combination of ARID1A and ß-Catenin meant a 2.209 fold increased risk for tumor-related death. This indicates that the ARID1A/ß-Catenin pathway connection might play a role in tumor progression in endometrioid endometrial carcinoma. It could already be demonstrated that ARID1A loss and ß-Catenin mutation, each seen individually, are important in progression of different types of human cancer [16,53,54]. Additional studies will have to be conducted to evaluate the association between ß-Catenin and the chromatin remodelling gene ARID1A in uterine endometrioid carcinoma further.

In the present study it was possible to identify three prognostic factors that showed differences in independent survival analysis. For the tumor suppressor gene p53 we were able to show that patients with overexpression or no expression of p53 had a worse overall survival compared to those with normal p53 expression. This is consistent with a number of studies determining the prognostic value of p53 in ovarian and uterine carcinoma [27,35] and in contrast to other studies [54,55].

It could be observed that nuclear and membrane/cytoplasm positive staining as well as nuclear and membrane negative staining of ß-Catenin were markers for worse overall survival which agrees with some studies [56,57] that show reduced ß-Catenin cell surface expression was a predictor for poor survival but not with other studies [58,59] that associate strong membranous β-Catenin expression with shorter progression free survival. Discordance between these results could be due to different patterns of ß-Catenin and p53 expression in ovarian and uterine carcinoma, especially in different staging and grading, and differences in the methods of immunohistochemical interpretation.

Similar to previous studies it could be determined that ARID1A loss in ovarian and uterine tumors is a predictor for poor survival [49,60–62]. ARID1A expression was also reported to be a prognostic marker for several other cancers such as gastric cancer, clear cell renal cell carcinoma and cervical cancer [63–65].

At this point some limitations of this study may be noted which can be addressed in future studies. They include mostly the limited numbers of cases within histological subgroups, especially clear cell carcinoma with a total number of only 17 cases. To prevent the effect, that ovarian and uterine carcinoma can be very heterogeneous in immunohistochemical interpretation the tissue microarray construction was done by using three core biopsies from each specimen. Nevertheless some cases might not have been adequately represented due to loss of cancer tissue material.

In summary, a significant association between AIRD1A and ß-Catenin expression was discovered in endometrioid uterine tumors. This suggests that concomitantly ARID1A negative staining and ß-Catenin nuclear and membrane/cytoplasm positive (n+m+) staining respectively ß-Catenin nuclear and membrane negative staining (n-m-) might play a role in tumor progression in type I endometrial cancer. Furthermore ARID1A, p53 and ß-Catenin turned out to be three promising prognostic factors showing significant differences in independent survival analysis, indicating that ARID1A, p53 and ß-Catenin could be used along traditional clinical and morphologic factors to predict the prognosis of patients with clear cell and endometrioid ovarian and uterine cancer.

## Supporting information

S1 Table Status of p53 according to tumor grade (N = 97). (DOCX)

S1 File Data set. (XLSX)

S1 Fig Status of ARID1A according to FIGO stage (N = 97). (TIF)
