# Supplementary material for: The ARID1A, p53 and ß-Catenin statuses are strong prognosticators in clear cell and endometrioid carcinoma of the ovary and the endometrium
Source: PLoS One. 2018 Feb 16;13(2):e0192881. doi: 10.1371/journal.pone.0192881 (PMC5815611; doi:10.1371/journal.pone.0192881)
Supplement: S1 Table — (DOCX) [file pone.0192881.s001.docx]

|  | p53 – or +++ | p53 + |
| --- | --- | --- |
| Expression N (%) | 36 (37.1) | 61 (62.9) |
| tumor grade – clear cell carcinoma | 12 (70.6) | 5 (29.4) |
| G1 | 0 | 0 |
| G2 | 0 | 0 |
| G3 | 12 (100) | 5 (100) |
| tumor grade – endometrioid carcinoma | 24 (17.5) | 56 (70.0) |
| G1 | 5 (20.8) | 22 (39.3) |
| G2 | 4 (16.7) | 25 (44.6) |
| G3 | 15 (62.5) | 9 (16.1) |
| p-value < 0.001 |  |  |
