# Supplementary figures and images for: The ARID1A, p53 and ß-Catenin statuses are strong prognosticators in clear cell and endometrioid carcinoma of the ovary and the endometrium
Source: PLoS One. 2018 Feb 16;13(2):e0192881. doi: 10.1371/journal.pone.0192881 (PMC5815611; doi:10.1371/journal.pone.0192881)

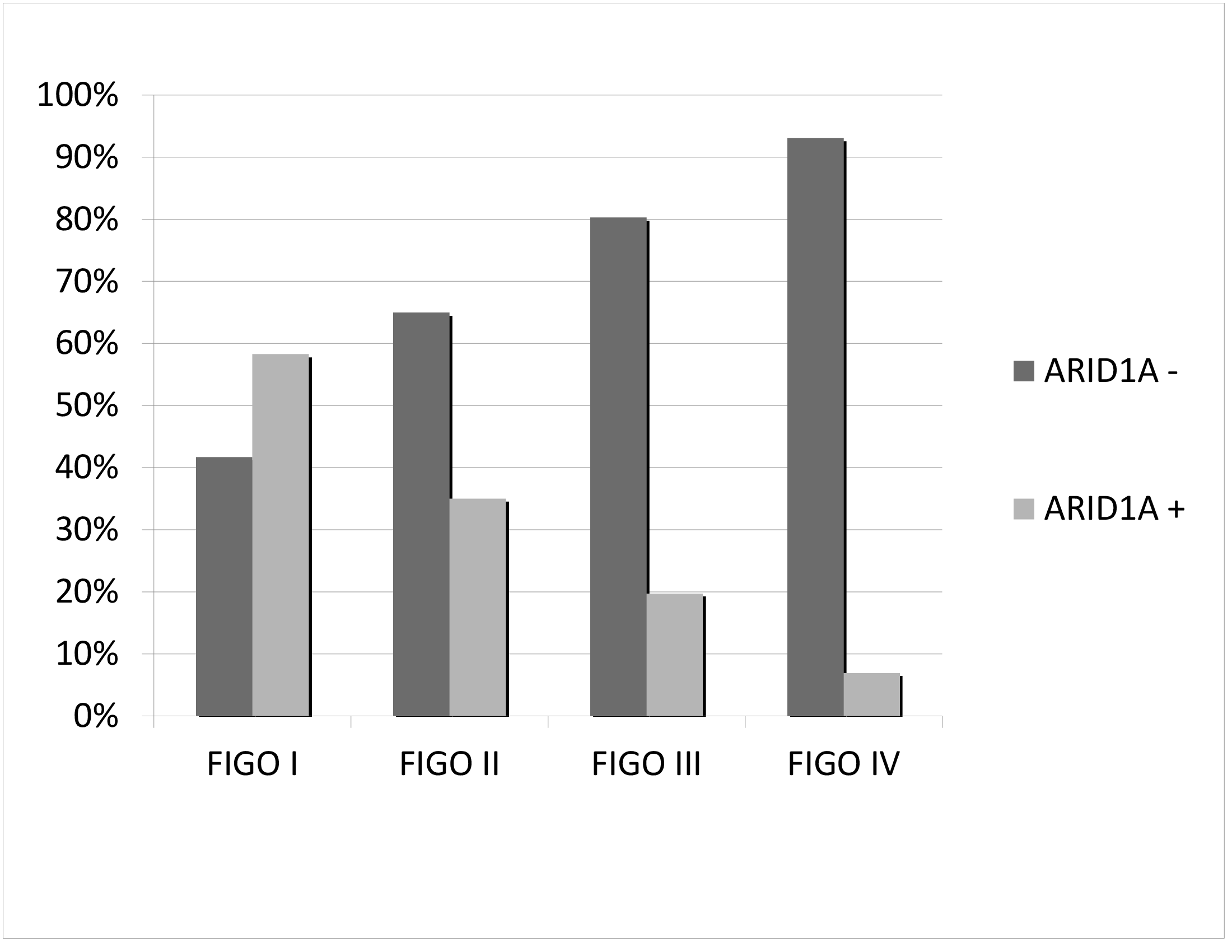

Supplement: S1 Fig — (TIF) [file pone.0192881.s003.tif]
